# Correlation between angioarchitectural characteristics of brain arteriovenous malformations and clinical presentation of 183 patients

**DOI:** 10.1590/0004-282X-ANP-2020-0291

**Published:** 2021-12-31

**Authors:** Ulysses Caus Batista, Benedito Jamilson Araujo Pereira, Andrei Fernandes Joaquim, Helder Tedeschi, Ronie Leo Piske

**Affiliations:** 1 Hospital Beneficência Portuguesa, Departamento de Neurorradiologia Intervencionista, São Paulo SP, Brazil. Hospital Beneficência Portuguesa Departamento de Neurorradiologia Intervencionista São Paulo SP Brazil; 2 Universidade Estadual de Campinas, Departamento de Neurocirurgia, Campinas SP, Brazil. Universidade Estadual de Campinas Departamento de Neurocirurgia Campinas SP Brazil

**Keywords:** Central Nervous System Vascular Malformations, Intracranial Arteriovenous Malformations, Seizures, Stroke, Malformações Vasculares do Sistema Nervoso Central, Malformações Arteriovenosas Intracranianas, Convulsões, Acidente Vascular Cerebral

## Abstract

**Background:**

The correlation between angioarchitecture and clinical presentation of brain arteriovenous malformation (bAVM) remains a subject of debate.

**Objective:**

The main purpose of the present study was to assess the correlation between angioarchitectural characteristics of bAVM and clinical presentation.

**Methods:**

A retrospective review of all consecutive patients presenting a bAVM who underwent a cerebral angiography at Beneficencia Portuguesa Hospital in São Paulo between January 2006 and October 2016 was carried out. Patients were divided in five groups: group 1 - hemorrhage; group 2 - seizure; group 3 - headache; group 4 - progressive neurological deficits (PND); group 5 - incidental).

**Results:**

A total of 183 patients were included, with group 1 comprising 56 cases, group 2 49 cases, group 3 41 cases, group 4 28 cases, and group 5 9 cases. Regarding hemorrhage presentation, a statistical correlation was observed with female gender (P < 0.02), Spetzler-Martin 3B (P < .0015), and lesions with low flow (P < 0.04). A positive association was found between group 2 and age less than 36 years (P < 0.001), male sex (P < 0.018), presence of superficial lesions not classified as SM 3B (P < 0.002), presence of venous ectasia (p <0.03), and arterial steal phenomenon (P < 0.03). Group 4 was associated with older age (P < 0.01).

**Conclusions:**

Angioarchitectural characteristics can be correlated with some clinical presentations as well as with some clinical data, making it possible to create predictive models to differentiate clinical presentations.

## RESUMO

**Antecedentes:**

A correlação entre a angioarquitetura e a apresentação clínica da Malformação Arteriovenosa do cérebro (MAVc) permanece um assunto de debate.

**Objetivos:**

Correlacionar as características angioarquiteturais das MAVc com a apresentação clínica.

**Métodos:**

Estudou-se pacientes consecutivos atendidos no Hospital Beneficência Portuguesa-SP, entre 2006 a 2016. Após análise geral, criaram-se cinco grupos de acordo com a apresentação clínica: 1- Hemorragia; 2 - Epilepsia; 3 - Cefaléia; 4 - Déficit Neurológico Progressivo (DNP) e 5 - Incidental. Características epidemiológicas (clínica e topografia) e angioarquiteturais (Classificação de Spetzler-Martin Modificada - SMM; Fluxo intranidal; Aneurismas arteriais, intranidais e venosos; Ectasia venosa; Congestão venosa; “Roubo” arterial; Vascularização dural; Drenagem Venosa Profunda) foram analisadas.

**Resultados:**

183 pacientes foram incluídos e analisados globalmente. Após essa etapa, foram divididos nos grupos: 1 - 56 casos (30,6%); 2 - 49 casos (26,7%); 3 - 41 casos (22,4%); 4 - 28 casos (15,3%) e 5 - 9 casos (4,9%). Principais achados foram referentes a apresentação hemorrágica, na qual observamos correlação estatística positiva com o sexo feminino (P<0,02), lesões classificadas como SMM 3B (P<0,0015) e baixo fluxo (P<0,04). Relacionado à epilepsia, encontramos significância estatística que possibilitou a correlação com pacientes com idade inferior a 36 anos (P<0,001), sexo masculino (P<0,018), lesões superficiais (P<0,002), presença de ectasia venosa (P<0,003) e “roubo” arterial (P<0,01). Pacientes com DNP se apresentam com idade superior aos demais (P<0,01).

**Conclusões:**

Após análise multivariada, foi possível separar as MAV em grupos de acordo com as características angioarquiteturais, comprovando que algumas dessas características estão fortemente relacionadas a determinada manifestação.

## INTRODUCTION

Brain arteriovenous malformations (bAVM) are rare lesions, with an estimated annual incidence of 1/100,000 new cases per year, accounting for about 2% of all hemorrhagic cerebrovascular onsets^1^. According to Ondra et al^2^, patients present an annual bleeding rate of 3.0%, with severe cumulative risk of morbidity (2.7%/year) and an annual mortality rate of 1%. In addition to the hemorrhage, these lesions may also cause epileptic seizures, headaches and/or progressive neurological deficits (PND). Lasjaunias et al suggested that these different forms of clinical presentations could be secondary to the location of the lesion and angioarchitectural characteristics of bAVM^3^. However, the correlation between angioarchitecture and bAVM clinical presentation remains a subject of debate^4^.

The main purpose of the present study was to assess the correlation of angioarchitectural characteristics of bAVM with the clinical presentation of patients at diagnosis.

## METHODS

After obtaining approval from the Institutional Review Board (No. 59830715.0.0000.5483), we conducted a retrospective database review of all consecutive patients presenting a bAVM who underwent a cerebral angiography of the six intracranial vessels following the same protocol at Beneficencia Portuguesa Hospital in Sao Paulo (Sao Paulo, Brazil) between January 2006 and October 2016. Clinical and radiological data were collected.

### Inclusion criteria

Inclusion criteria were patients aged ≥18 years, with complete clinical, epidemiological, and angiographic data on hospital admission charts who were diagnosed with bAVM. All angiographies were performed by the lead author (RLP).

### Exclusion criteria

Exclusion criteria were patients aged <18 years and those with incomplete data on admission charts. Other types of intracranial arteriovenous malformations (AVM) were excluded. In addition, patients who received any other prior treatment, such as surgery, embolization or radiosurgery, and therefore had alterations in the original AVM angioarchitecture, were also excluded. 

### Epidemiological characteristics

The following data were extracted from the database: age, sex, clinical presentation, and lesion topography.

*Clinical presentation*

Patients were classified into 5 groups according to the chief complaint that led to the examination: 1) intracranial hemorrhage, 2) seizures 3) persistent headache, 4) progressive neurological deficits (PND), and 5) incidental finding on image exam.

*Topography*

Lesions related to the cerebral cortex were classified as superficial AVMs, regardless of the lobe in which they were located or if they were corticoventricular. Malformations located in the basal ganglia (with exclusive nutrition of perforating branches) were classified as deep lesions. Those located in the cerebellum or in the brainstem were classified as posterior fossa AVMs^5^. 

### Angioarchitectural characteristics 

The following angioarchitectural characteristics (Table 1) were analyzed:

**Table 1.** Description of demographic and angioarchitectural characteristics analyzed in this study.

Characteristics studied	Description
Clinical presentation	Five groups were created: 1) intracranial hemorrhage, 2) epileptic seizures, 3) persistent headache, 4) progressive neurological deficits, 5) incidental finding on image exam.
Topography	Lesions related to cerebral cortex were classified as superficial AVMs; in the basal ganglia (exclusive nutrition by perforating branches) as deep lesions. Those located in the cerebellum or in the brainstem were classified as posterior fossa AVMs^5^.
Grade 3 Spetzeler-Martin scale modified	Grade 3 Spetzeler-Martin Scale was divided into two groups: grade 3A and 3B. AVMs that received two points for medium size, one point for eloquent location, and zero for superficial venous drainage were classified as 3A. Those that were small (1 point), located in eloquent areas (1 point), and a deep venous drainage (1 point) were classified as 3B (Figure 1).
Intralesional flow	Classified as high or low flow. High flow referred to cases where contrast opacification occurred only in the malformation without filling other normal branches of this territory. In low-flow lesions, other arteries of the same vascular territory were also opacified.
Intranidal aneurysms	Aneurysmal formation located inside the nidus and confirmed in more than one angiographic projection (Figure 2-A).
Venous aneurysms	Aneurysmatic dilatations in a vein draining the lesion, confirmed by different angiographic projections (Figure 2-B).
Venous ectasia	Marked increase in the diameter and tortuosities of the vessel that drains the AVM (Figure 2-B)^8^.
Venous congestion	Redirecting draining flow to other veins hindering normal brain-tissue drainage (Figure 2-C).
Arterial steal	Insufficient filling of the normal branches at the same territory where the AVM are located.
Dural vascularization	Participation of dural vessels supplying the AVM (Figure 2-D).
Deep venous drainage	Direct drainage to the deep venous system (Figure 3)^9^.

*Modified Spetzler-Martin grading scale*

**Figure 1.**
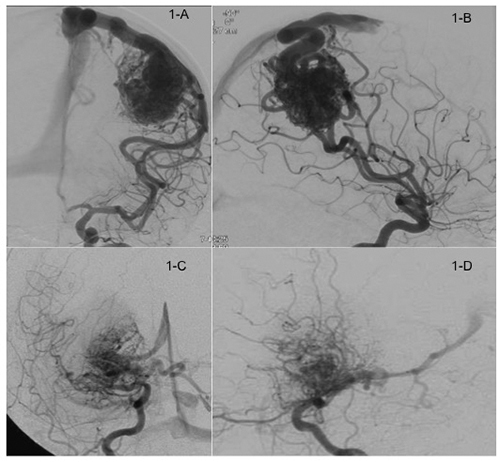
A: Left internal carotid artery angiogram, late arterial phase (frontal view), showing a medium (3 - 6 cm) AVM in a superficial location with exclusive superficial venous drainage. B: Left internal carotid artery angiogram, late arterial phase (lateral view), of the same AVM in 1A, which was classified as 3A. C: Right internal carotid artery angiogram, late arterial phase (frontal view), showing a small (less than 3 cm) AVM in the basal ganglia (deep location) with deep venous drainage; D: Right internal carotid artery angiogram, late arterial phase (lateral view) of the same AVM in 1C, which was classified as 3B.

Although the literature usually follows the Spetzler-Martin Grading Scale (S-M)^6^, we considered the modifications proposed by Oliveira et al^1^, who sub classified bAVM grade 3 of the S-M. In this study, we divided bAVM grade 3 into two groups: modified S-M 3A and S-M 3B. The bAVMs that received a total of three points for medium size (2 points), eloquent location (1 point) and only superficial venous drainage (0 points) were classified as 3A, whereas those that were small (1 point), located in eloquent areas (1 point), and a deep venous drainage (1 point) were classified as 3B. In Figure 1, we present an example of AVM grades 3A and B. 

*Intralesional flow*

BAVMs were classified as high- or low-flow. High-flow referred to cases where opacification after contrast injection occurred only in the malformation without filling other normal branches of this territory. In low-flow lesions, other arteries of the same vascular territory were also opacified. 

*Intranidal aneurysms*

**Figure 2.**
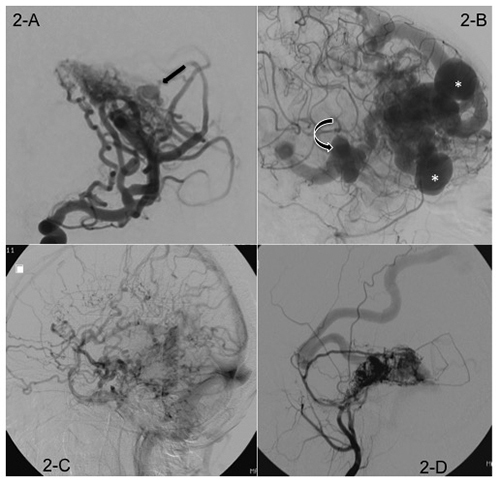
A: Left internal carotid artery angiogram, arterial phase in oblique view, highlighting an intranidal aneurysm (arrow); B: Left internal carotid artery, venous phase in lateral view, demonstrating two venous aneurysms (asterisk) and venous ectasia (curved arrow); C: Left internal carotid artery, late venous phase in lateral view, showing a venous congestion; D: Right external carotid angiogram, arterial phase in lateral view, demonstrating the dural vascularization of the AVM.

These are aneurysmal formations located inside the nidus. All intranidal aneurysms were confirmed in more than one angiographic projection (Figure 2-A).

*Arterial aneurysms (not intranidal)*

Aneurysms not directly related to the nidus of the AVM were divided into two: flow-related aneurysms, which refer to the location at the arterial pathway supplying the AVM, and aneurysms not related to the main supply for the AVM^7^.

*Venous aneurysms*

These are localized aneurysmatic dilatations in a vein draining the lesion, which were confirmed by different angiographic projections (Figure 2-B). 

*Venous ectasia*

This refers to a marked increase in the diameter of the vessel that drains the AVM associated with tortuosities (Figure 2-B)^8^.

*Venous congestion*

This is the case when, in addition to the anatomically expected venous drainage, the flow is redirected to other veins, obstructing the normal drainage of brain tissue (Figure 2-C).

*Arterial steal*

This is related to a lack of filling of the normal branches of the same area where the AVM is located. These branches present retrograde filling by pial anastomoses, which could also supply the nidus.

*Dural vascularization*

This describes a situation in which the AVM is also supplied by dural vessels (Figure 2-D).

*Deep venous drainage*

**Figure 3.**
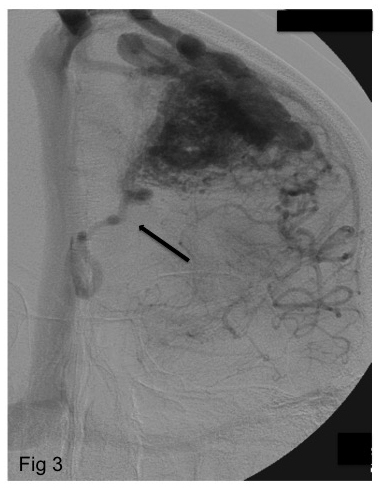
Left internal carotid angiogram, capillary phase (frontal view), showing a superficial AVM with deep venous drainage promoted by only one vessel (arrow). Although the major AVM drainage is done by superficial vessels, according to Spetzler-Martin Classification, the presence of this deep channel of drainage classifies this malformation as having deep venous drainage.

All bAVMs presenting direct drainage to the deep venous system were included in this group (Figure 3)^9^.

### Image acquisition

All patients underwent a complete angiographic study, which consisted in the analysis of the internal and external carotid arteries and vertebral arteries, in at least anteroposterior and lateral views. Other views were analyzed to disclose arterial or venous stenosis or aneurysms. The angiographic equipments used were: Philips Integris biplane (between 2006 and 2014) and Philips Allura Xper FD biplane (between 2014 and 2016). Images were stored in the Aurora PACS system version 1.6.7. UCB and RLP researchers analyzed all images together. 

### Statistical analysis

Data were analyzed using the following softwares: SPSS V17, Minitab 16, and Excel Office 2010. Statistical resources used were equality of proportions test, Chi-Square test, odds ratio, and multivariate analysis by logistic regression. The logistic regression models were confirmed by the Pearson, deviance, and Hosmer-Lemeshow tests. The level of significance was set at p < 0.05. 

## RESULTS

### Epidemiological analysis

A total of 183 patients were included with 93 (50.8%) males. The mean age of onset was 37 years (ranging from 18 to 84 years; SD: ±14.0). 

Cases were divided into five groups according the clinical presentation at diagnostic: group 1 (hemorrhage): 56 cases (30.6%); group 2 (seizure): 49 cases (26.7%); group 3 (headache): 41 cases (22.4%); group 4 (Progressive neurological deficits): 28 cases (15.3%), and group 5 (Incidental): 9 cases (4.9%).

Regarding the location of the lesions, superficial AVMs were the most common, with 124 cases (67.7%), followed by the deep AVMs, with 36 cases (18.6%) and by lesions located in the posterior fossa, with 23 cases (12.5%). Table 2 summarizes these findings and the results for each group.

**Table 2.** Distribution of 183 patients according to demographic characteristics.

Demographic characteristics	General	%	Hemorrhage	%	Seizure	%	Headache	%	PND	%	Incidental	%
Mean age	37		37,5		32		34,5		45,2		45,4	
Maximum age	84		75		55		69		71		84	
Minimum age	18		18		18		18		22		19	
Standat deviation	14		12		10,3		13,5		16,2		22,5	
Male	93	50.8	22	39.2	31	63.2	18	43.9	17	60.7	5	55.5
Female	90	49.2	34	60.7	18	36.8	23	56.1	11	39.3	4	44.5
Superficial lesion	124	67.7	25	44.6	46	93.9	32	75.6	17	60.7	4	44.4
Deep lesion	36	18.6	19	33.9	3	6.1	6	14.6	6	21.4	2	22.2
Posterior fossa lesion	23	12.5	12	21.4	0	0	3	7.3	5	17.8	3	33.3
Total	183		56		49		41		28		9	

When comparing groups, a difference was found in the mean age of symptom onset between groups 2 and 4 (P < 0.01) and 3 and 4 (P < 0.01). The other groups did not differ in the mean age of onset.

Considering sex, a higher prevalence of bleeding was found in women while a higher prevalence of seizures was found in men (P < 0.014). There was no statistical difference between sexes in the other groups.

The topography of the AVMs was also studied within each group, comparing the three possible locations, as shown in Table 1. There was a correlation between hemorrhage and deep lesions or lesions in the posterior fossa compared to superficial lesions (P < 0.0003 and P < 0.002, respectively). An important difference was also observed in group 2, in which patients with superficial AVMs had a higher incidence of epilepsy compared to patients with deep lesions (P < 0.002). Differences were also observed between superficial and deep or posterior fossa AVMs in group 4, showing that superficial lesions presented more PND than lesions with other topographies (P < 0.007 and P < 0.01, respectively). No statistical differences were observed among patients who presented headaches. Table 3 summarizes all these findings.

**Table 3.** General and group distribution of the 183 MAVs in relation to the angioarchitectural characteristics studied.

Angioarchitectural characteristics	General	%	Hemorrhage	%	Seizure	%	Headache	%	PND	%	Incidental	%
SM 1	47	25.6	16	28.5	11	22.4	10	24.4	7	25	3	33.3
SM 2	61	33.3	13	23.2	17	34.6	21	42.8	10	35.7	2	22.2
mSM 3A	30	16.3	7	12.5	11	22.4	8	19.5	4	14.3	1	11.1
mSM 3B	30	16.3	17	30.3	2	4	3	7.3	6	21.4	2	22.2
SM 4	13	7.1	3	5.3	8	16.3	0	0	1	3.6	1	11.1
SM 5	2	1	0	0	1	2	0	0	1	3.6	0	0
High flow	65	35.5	13	23.2	20	40.8	19	46.3	11	39.2	2	22.2
Intranidal aneurysms	41	22.4	16	28.5	10	40.8	6	14.6	5	17.8	4	44.4
Venous aneurysms	30	16.3	5	8.9	8	16.3	10	24.4	5	17.8	2	22.2
Venous ectasias	26	14.2	5	8.9	12	24.5	2	4.9	7	25	0	0
Venous congestions	39	21.3	9	16	9	18.3	10	24.4	9	32.1	2	22.2
Arterial steal phenomenon	6	3.2	0	0	4	8.1	1	2.4	0	0	1	11.1
Dural vascularization	13	7.1	4	7.1	5	10.2	3	7.3	0	0	1	11.1
Deep venous drainage	64	34.9	25	44.6	13	26.5	9	22	13	46.4	4	44.4
Total	183		56		49		41		28		9	

The results of the epidemiological analysis of each group studied are presented:

Group 1 (hemorrhage) - 56 patients: The mean age of this group was 37.5 years (ranging from 18 to 75 years; SD: ± 11.97), with 22 (39.2%) males. In 24 (42.8%) cases, the lesion had a superficial location, in 19 cases (33.9%) AVMs were deep and in the remaining 12 cases (21.4%) AVMs were located in the posterior fossa. Deep and posterior fossa AVMs presented a higher incidence of hemorrhage than superficial AVMs (P < 0.01).

Group 2 (seizure) - 49 patients: The mean age of this group was 32 years (ranging from 18 to 55 years, SD: ± 10.37) and 31 (63.2%) were male. In 46 (93.8%) cases, lesions were located superficially, in three patients (6.2%) AVMs were deeply located, and no patient who presented with epilepsy had lesions in the posterior fossa. Superficial AVMs had a higher incidence of epilepsy compared to deep AVMs (P < 0.01).

Group 3 (headache) - 41 patients: Patients in this group had a mean age of 34.5 years (ranging from 18 to 69 years, SD: ± 13.5), 18 (44%) of whom were male. The main location was superficial in 32 cases (78%), followed by deep lesions with 10 (14.6%) patients, and those located in the posterior fossa with seven (7.3%) cases. There was no statistical difference between lesions locations in this group.

Group 4 (progressive neurological deficits) - 28 patients: The mean age in this group was 45.2 years (range 22-71, SD: ± 16), and 17 (60.7%) were male. Superficial lesions were the most frequent, with 17 (60.7%) patients, followed by the deep AVMs and AVMs in the posterior fossa, with six (21.4%) and five (17.8%) cases, respectively. The superficial location correlated with neurological deficits compared to the other locations in this group (P < 0.01).

Group 5 (incidentals): 9 patients: Patients had a mean age of 45.4 years (ranging from 19-84 years; SD: ± 22.5), with the male sex accounting for five (55.5%) patients. The AVM were superficial in four (44.4%) cases, deep in two (22.2%) and located in the posterior fossa in three (33.3%) cases.

### Angioarchitectural analysis

The angioarchitectural characteristics of 183 bAVM were analyzed. The results for bAVMs as a total group and by subgroup are summarized in Table 3.

*Modified Spetzler-Martin classification*

AVM classified as SM 3B had a higher risk of bleeding than the other classifications (P < 0.0015, OR: 3.82, 95% CI, 1.70-8.57). In addition, patients in the epilepsy group were less likely to have a deep AVM (P < 0.01; OR: 3.8; CI: 1.70 - 8.57 ). 

*Study of AVM flow velocity*

Lesions with low flow were more susceptible to hemorrhage (P < 0.032; OR: 2.29; CI: 1.12 - 4.68) compared to the other groups.

*Presence of intranidal aneurysms*

The presence of intranidal aneurysms in AVM did not correlate with any clinical presentation (P = 0.22).

*Arterial aneurysms*

High-flow aneurysms and aneurysms not related to high-flow had an incidence of 22.4% and 7.1%, respectively. The combination of cerebral aneurysm and AVM had an incidence of 29.5%.

*Venous aneurysms*

There was no statistical correlation between the presence of venous aneurysms and the clinical presentation of patients (P = 0.11).

*Venous ectasia*

The presence of venous ectasia did not show statistical correlation with hemorrhage (P = 0.31; OR: 0.52; 95% CI: 1.18-1.47). However, it was statistically significant in the epilepsy group (P = 0.03; OR: 2.77; 95% CI: 1.18 - 6.53)

*Venous congestion*

The presence of venous congestion was not significantly correlated with clinical presentation (P = 0.34).

*Arterial “steal”*

Arterial “steal” was significantly correlated with epilepsy (P = 0.02).

*Dural vascularization*

There was no statistical significance between dural vascularization and the clinical presentation (P = 0.76).

*Deep venous drainage (DVD)*

There was a correlation trend for DVD in the hemorrhage group (P < 0.08).

Table 3 summarizes the results of the univariate analysis of angioarchitectural characteristics per group of patients.

*Correlation between high-flow AVM and other angioarchitectural characteristics*

Lesions with a high flow had a higher prevalence of intranidal aneurysms, venous ectasia, venous congestion and arterial “steal", as summarized in Table 4.

**Table 4.** Univariate analysis of the AVM distribution according to the angioarchitectural characteristics and topography.

Angioarchitectural characteristics	Hemorrhage			Seizure			Headache			PND		
	P	OR	CI	P	OR	CI	P	OR	CI	P	OR	CI
Superficial x deep location	**0.0003**	0.22	0.10 - 0.49	**0.002**	6.48	1.88 - 22.34	0.361	1.73	0.66 - 4.56	**0.0007**	7.72	2.42 - 24.62
Superficial x PF location	**0.0028**	0.23	0.09 - 0.585	0	0	0	0.29	2.31	0.64 - 8.32	**0.012**	5.56	1.59 - 19.37
Deep x PF location	0.82	1.02	0.35 - 2.92	0	0	0	0.995	1.33	0.29 - 5.95	0.8845	0.72	0.19 - 2.70
Grade 3B mSM scale	**0.0015**	3.82	1.70 - 8.57	-	-	-	-	-	-	-	-	-
Low flow	**0.032**	2.29	1.12 - 4.68	0.46	1.36	0.69 - 2.67	0.144	1.8	0.88 - 3.65	0.811	1.21	0.52 - 2.76
Intranidal aneurysms	0.25	1.63	0.78 - 3.37	0.848	0.85	0.381 - 1.90	0.25	0.52	0.20 - 1.35	0.7	0.71	0.25 - 2.02
Venous aneurysms	0.11	0.4	0.14 - 1.10	0.83	0.99	0.41 - 2.40	0.18	1.96	0.83 - 4.62	0.96	1.13	0.39 - 3.25
Venous ectasias	0.31	0.52	0.18 - 1.47	**0.03**	2.77	1.18 - 6.53	0.1	0.26	0.05 - 1.17	0.109	2.53	0.94 - 6.80
Venous congestions	0.34	0.61	0.27 - 1.40	0.7	0.78	0.34 - 1.78	0.74	1.25	0.55 - 2.85	0.204	1.97	0.81 - 4.79
Arterial "steal"	0	0	0	**0.02**	5.86	1.03 - 33.11	0.87	0.68	0.07 - 6.03	0	0	0
Dural vascularization	0.76	1.00	0.29 - 3.42	0.21	2.38	0.77 - 7.29	0.77	1.04	0.27 - 3.97	0	0	0
Deep venous drainage	0.087	1.79	0.94 - 3.44	0.191	0.58	0.28 - 1.19	0.08	0.45	0.20 - 1.03	0.25	1.75	0.77 - 3.95

After univariate analysis, a multivariate logistic regression study was performed to evaluate the possibility of creating models that could predict the studied clinical presentations. We found a positive correlation between hemorrhage and female sex (P < 0.02), AVM S-M 3B (P < 0.001), and low flow (P < 0.04). 

In patients with epilepsy, we observed an association with age less than 36 years (P < 0.001), male sex (P < 0.018), superficial lesions not classified as SM 3B (P < 0.002), presence of venous ectasia (P <0.03), and arterial “steal” phenomenon (P < 0.03). Predictive models could not be generated for the other groups. Table 5 summarizes these findings.

**Table 5.** Multivariate analysis for the creation of predictive models for the clinical presentations studied (hemorrhage and epilepsy).

Multivariate analysis	Hemorrhage		Seizure	
	P	OR (CI 95%)	P	OR (CI 95%)
Mean age			0.001	0.95 (0.92 - 0.98)
Male	0.021	0.45 (0.23 a 0.89)	0.018	2.46 (1.17 a 5.19)
S-M 3B	0.001	3.95 (1.69 a 9.2)	0.002	0.08 (0.01 a 0.4)
High flow studied	0.047	0.47 (0.22 a 0.99)		
Intranidal aneurysm				
Venous aneurysm				
Venous ectasia			0.037	2.84 (1.07 a 7.56)
Venous congestion				
Arterial steal phenomenon			0.039	2.3 (1.04 a 5.1)
Dural vascularization				
Deep venous drainage				

## DISCUSSION

BAVMs are rare lesions, but they may have serious clinical consequences, such as intracranial hemorrhage^1^^,^^8^ that result in severe morbidity and even death^2^. In addition to hemorrhage, epileptic seizures, persistent headaches, and progressive neurological deficits are also associated with these lesions, which increase morbidity in these patients. Considering these points, evaluation of the AVM angioarchitecture is fundamental for the management of these patients. Tong et al found that female sex correlated with hemorrhagic presentations, whereas men had a higher risk of epileptic seizures, which was similar to our findings^10^.

The mean age of symptom onset of our patients was similar to those found in the literature^11^^-^^14^. However, pediatric patients are commonly included, which may affect the age of onset of patients with AVM. Hetts et al only studied adult patients, reporting a mean age of clinical symptoms of 42.6 years for patients with AVM^15^, slightly higher than our sample. We did not find studies that grouped the age of clinical presentation of AVM according to clinical presentation. In our study, we observed that PND patients were more advanced in age at the onset of symptoms than those patients with epilepsy or headaches *(P* < .01). However, with regard to age, patients with hemorrhagic AVMs did not show any differences relative those from the other groups. 

According to the literature^16^^-^^18^, deep AVMs have a higher rate of hemorrhage. Lesions classified as S-M 3B were also more related to hemorrhagic events than other lesions (P < 0.01). The topography of the lesions was also studied within each group, showing that patients who had hemorrhagic events had deep or posterior fossa lesions (P < 0.003 and P < 0.00028, respectively), with no differences between these two presentation sites (P > 0.82). In patients with epilepsy, lesions were predominantly superficial (P < 0.002), as well as in patients with DNP, where superficial lesions predominated compared to deep-seated lesions or lesions located in the posterior fossa (P < 0.0007 and P < 0.01, respectively). This is probably a causative phenomenon, that is, the superficial location may increase the chances of cortical irritation with gliosis, leading to epileptic seizures^6^.

Kim et al^19^ and Duong et al^20^ studied predictive factors for hemorrhage in patients with AVMs, finding a correlation between exclusive deep-vein drainage and hemorrhagic presentation. However, according to Spetzler et al^6^, deep-vein drainage is characterized by at least one deep vessel. By considering lesions with exclusive deep-vein drainage, we found similar results (P < 0.0015). However, if we maintained the original S-M classification of DVD, we found only a tendency for hemorrhage in patients with this type of drainage (P < 0.087). We believe that deeply located AVMs are more prone to hemorrhage and that the majority of deep-seated lesions cause deep-vein drainage. 

 Kubalek et al also reported that low-flow AVMs had a higher risk of hemorrhage, similar to our findings (P > 0.03, OR 2.29, 95% CI 1.12-4.68)^12^. Of note, the majority of AVMs that present bleeding are deep-seated, and high flow is not commonly found in that location. 

 Stapf et al reported a positive correlation between the presence of intranidal aneurysms and hemorrhagic events.^21^ However, Pollock et al^22^, in a study of 313 patients, did not report any relationship between the presence of intranidal aneurysm and hemorrhage, which was similar to our findings. Intranidal aneurysms were more frequently observed in lesions with high flow, suggesting that they are a secondary event, as observed in our series. Mast et al studied the correlation between arterial steal and DNP, and found no statistical significance between these characteristics^23^. However, we found this correlation in patients who presented epilepsy (P = 0.02). It should be noted that, similar to intranidal aneurysms, this phenomenon is more prevalent in lesions with a high flow, which are also more commonly found in patients with epilepsy (P < 0.03, OR 10, 95% CI 1.14-87.5).

Redekop et al and Kubalek et al reported that the incidence of hyperflow aneurysms in patients with bAVMs were 15.3% and 12.3%, respectively^7^^,^^12^. In these two studies, it was found that the association of aneurysm and AVMs had an incidence of hemorrhage of about 7%/year^7^. In our sample, we found a high incidence of these aneurysms (22.4% of hyperflow aneurysms and 7.1% of non-hyperflow aneurysms).

Shankar et al studied the effects of venous ectasia on cerebral AVMs, and, similar to our findings, observed that the ectasied drainage vessels showed a positive correlation with epilepsy (P < 0.03, OR 2.77, 95% CI, 1.18-6.53)^13^. 

Pan et al reported that vascularization by perforating arteries and exclusive deep-vein drainage presented a higher rate of hemorrhage^17^. It is interesting to note that in the study, the deep location of AVMs did not present positive results, even though the vascularization of perforating arteries and exclusive deep-vein drainage are strongly related to this location. Kandai et al and Stefani et al also reported that the deep location of lesions is the main predictive characteristic for bleeding events^16^^,^^18^. In our study, the predictive model for hemorrhage showed that female gender (P < 0.02), lesions classified as modified S-M 3B (small, deep, and with exclusively deep-vein drainage) (P < 0.001) and low-flow AVMs (P < 0.047) were associated with high risk of hemorrhage. These results are interesting, because they differ from those of epilepsy-related lesions, which have stronger association with younger age (P < 0.004), male sex (P < 0.03), superficial location (P < 0.002), presence of venous ectasia (P < 0.03) and arterial steal phenomenon (P < 0.03). 

We concluded that angioarchitectural characteristics of bAVMs may be correlated with some clinical presentations as well as with some clinical data. Hemorrhagic events were associated with female sex, deep location, and low-flow AVMs. Epilepsy presentation was associated with younger age, male sex, superficial location, presence of ectasied veins, and arterial steal phenomenon. Posterior fossa lesions were not correlated with epilepsy in our series, and there was more frequent observation of PND in older patients.
